# Intervortex Venous Anastomosis in the Macula in Central Serous Chorioretinopathy Imaged by En Face Optical Coherence Tomography

**DOI:** 10.3390/jcm12062088

**Published:** 2023-03-07

**Authors:** José Ignacio Fernández-Vigo, Daniela Rego-Lorca, Francisco Javier Moreno-Morillo, Bárbara Burgos-Blasco, Alicia Valverde-Megías, Carmen Méndez-Hernández, Lorenzo López-Guajardo, Juan Donate-López

**Affiliations:** 1 Ophthalmology Department, Hospital Clínico San Carlos, Institute of Health Research (IdISSC), 28040 Madrid, Spain; 2 Centro Internacional de Oftalmología Avanzada, 28010 Madrid, Spain; 3 Ophthalmology Department, Hospital Infanta Sofía, San Sebastián de los Reyes, 19171 Madrid, Spain

**Keywords:** central serous chorioretinopathy, choroidal vessels, en face optical coherence tomography, intervortex veins anastomosis, pachychoroid disease

## Abstract

Purpose: To assess the presence of macular intervortex venous anastomosis in central serous chorioretinopathy (CSCR) patients using en face optical coherence tomography (EF-OCT). Methods: A cross-sectional study where EF-OCT 6 × 6 and 12 × 12 mm macular scans of patients with unilateral chronic CSCR were evaluated for anastomosis between vortex vein systems in the central macula. The presence of prominent anastomoses was defined as a connection with a diameter ≥150 µm between the inferotemporal and superotemporal vortex vein systems which crossed the temporal raphe. Three groups were studied: CSCR eyes (with an active disease with the presence of neurosensorial detachment; n = 135), fellow unaffected eyes (n = 135), and healthy eyes as controls (n = 110). Asymmetries, abrupt termination, sausaging, bulbosities and corkscrew appearance were also assessed. Results: In 79.2% of the CSCR eyes there were prominent anastomoses in the central macula between the inferotemporal and superotemporal vortex vein systems, being more frequent than in fellow eyes and controls (51.8% and 58.2% respectively). The number of anastomotic connections was higher in the affected eye group (2.9 ± 1.8) than in the unaffected fellow eye group (2.1 ± 1.7) and the controls (1.5 ± 1.6) (*p* < 0.001). Asymmetry, abrupt terminations and the corkscrew appearance of the choroidal vessels were more frequent in the affected eyes, although no differences in sausaging or bulbosities were observed. Conclusions: Intervortex venous anastomoses in the macula were common in CSCR, being more frequently observed in affected eyes than in fellow unaffected eyes and healthy controls. This anatomical variation could have important implications concerning the pathogenesis and classification of the disease.

## 1. Introduction

Central serous chorioretinopathy (CSCR) is one of the main causes of visual function impairment in working age patients. It is characterized by the presence of subretinal fluid (SRF) and could be associated with serous retinal pigment epithelium (RPE) detachment in the macula and the posterior pole [1].

The exact cause of CSCR is not fully understood, but it is believed to be related to choroidal vessel dilation and hyperpermeability, hence CSCR is included among the newly described pachychoroid diseases [2,3,4]. In this group of conditions, the abnormally dilated choroidal vessels produce direct compression of the overlying choriocapillaris (CC) [5,6]. This compression can alter the normal barrier function of the complex between the CC–RPE, leading to the accumulation of fluid. Abnormalities in the flow patterns, such as impaired or irregular flow of the CC and choroid, have been observed in CSCR patients corresponding to indocyanine green angiography (ICGA) anomalies [7,8,9,10,11,12,13].

Recently developed, en face optical coherence tomography (EF-OCT) is a rapid and non-invasive high-resolution imaging technique that allows for the obtaining of coronal images of the retina and choroid (C-Scans). Dansingani et al., using this technology, described common morphological features in the choroidal vessels of eyes with pachychoroid spectrum-related disorders, including the presence of dilated vessels with a distinctive morphology [14]. In addition, different characteristics and the appearance of the choroidal vessels have been described to be associated with CSCR eyes, such as an asymmetry of the choroidal vessel [15], an abrupt termination [14], or the presence of sausaging and bulbosities [16].

Accumulating evidence suggests that the presence of pachychoroid is possibly caused by vortex vein congestion leading to a remodeling to create new choroidal drainages routes via intervortex venous anastomosis [11,17,18,19]. Spaide et al. reported that intervortex venous anastomoses are common in pachychoroid eyes, including those with CSCR [20]. However, to date, there are no studies evaluating the presence of macular choroidal anastomoses in a large population of CSCR patients by means of EF-OCT.

Hence, the objective of the present study is to assess the presence of intervortex venous anastomosis in the macula in CSCR patients by EF-OCT, as well as other characteristics of the choroidal vessels, comparing affected CSCR with the fellow unaffected eyes and with healthy controls.

## 2. Materials and Methods

A cross-sectional study including 135 patients with unilateral chronic CSCR recruited among those who were attended over the period from January 2020 to May 2022, in Hospital Clínico San Carlos, Madrid (Spain) was conducted.

To participate in the study, individuals had to meet all of the inclusion criteria and none of the exclusion criteria, and to provide written informed consent. The study was approved by the Center’s Institutional Review Board, and the study protocol adhered to the tenets of the Declaration of Helsinki. The CSCR diagnosis was conducted in a multimodal imaging exploration based on the newly proposed criteria [1].

Both eyes of each patient were included for the assessment of the presence of macular anastomosis of the intervortex veins. Three groups were studied: CSCR eyes (n = 135), unaffected fellow eyes (n = 135), and eyes of healthy controls (n = 110).

Inclusion criteria for the chronic CSCR eyes was the persistence of SRF for ≥4 months and no previous treatments. For the unaffected fellow eyes, no SRF or retinal pigment epithelium detachment could be present, or previously detected, in order to be included.

Concerning the control group, the inclusion criteria were: age 18 years or older, healthy Caucasian subjects, a refractive error between +3.0 and −3.0 diopters, and an intraocular pressure (IOP) of less than 21 mmHg in both eyes.

Exclusion criteria for all eyes included were: any other ocular pathology, systemic pathology that typically affects the vessels such as diabetes, arterial hypertension or cardiac disorders, significant opacity of the lens or cornea that hinders obtaining good quality images, any previous ocular surgery or ocular treatment in the previous three months.

On the same day, recruited subjects underwent a medical history and a comprehensive ophthalmological examination, including OCTA imaging after pupil dilation. The examination included visual acuity (VA), refraction, slit-lamp biomicroscopy, and posterior segment ophthalmoscopy. The sex and age of each participant were recorded.

### 2.1. OCT and OCTA Examination

The device employed for the scans was a Plex Elite (Zeiss Meditec, Dublin, CA, USA) using the AngioPlex Elite 9000 algorithm (AngioPlex Elite 9000, Zeiss, Germany). This device is based on swept-source OCTA (SS-OCTA) technology and uses a central wavelength between 1060 nm and 1040 nm with scanning speed of 200,000 A-scans per second that offers a transverse resolution of 20 μm and an axial resolution of 6.3 μm.

The OCTA images were obtained by two experienced examiners (JIFV and DRL). Only images of good quality, as determined by a signal quality > 7/10, were included in the study, being analyzed after applying the device’s software projections removal algorithm and checking for appropriate segmentation.

**Figure 1:**
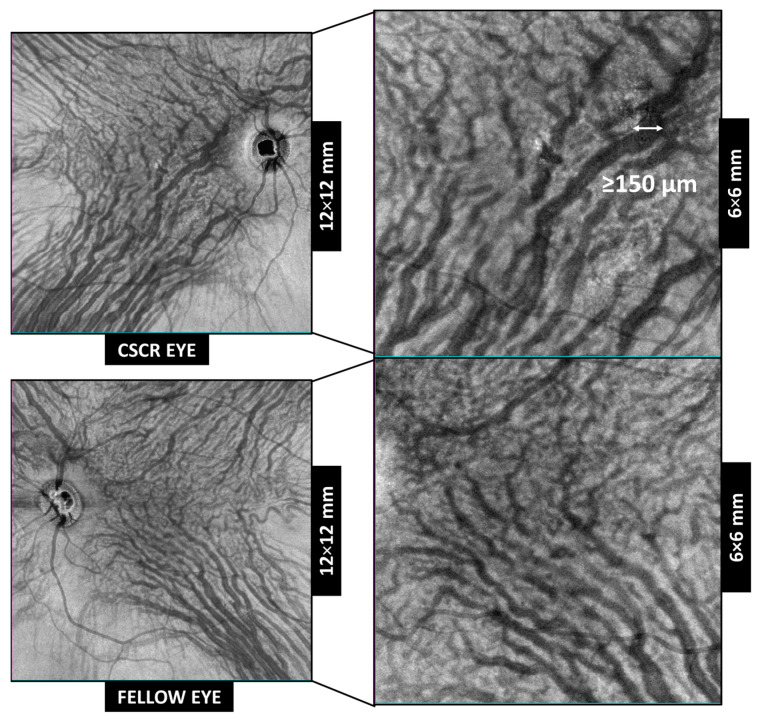
En face optical coherence tomography of the macular region used to analyze the presence of intervortex veins anastomosis in a cube scan of 6 × 6 mm and 12 × 12 mm in central serous chorioretinopathy (CSCR) eyes (upper row) and fellow unaffected eyes (bottom row).

The 6 × 6 mm and 12 × 12 mm scans centered on the macula were performed, and the SS-OCTA device was used to obtain the EF-OCT images (Figure 1). The small scan was used to measure the anastomosis with higher precision. For analysis, the image that corresponded to the choroid segmentation and was focused on the pachyvessels mainly in Haller’s layer was selected. The automatic segmentation of the software by default, selecting the legacy mode, produced a slab 100 µm beneath the RPE and a thickness slab of 100 µm. This was used as a reference. However, due to the individual variability of the choroidal thickness and Haller’s layer position in each subject, segmentation was individualized.

**Figure 2:**
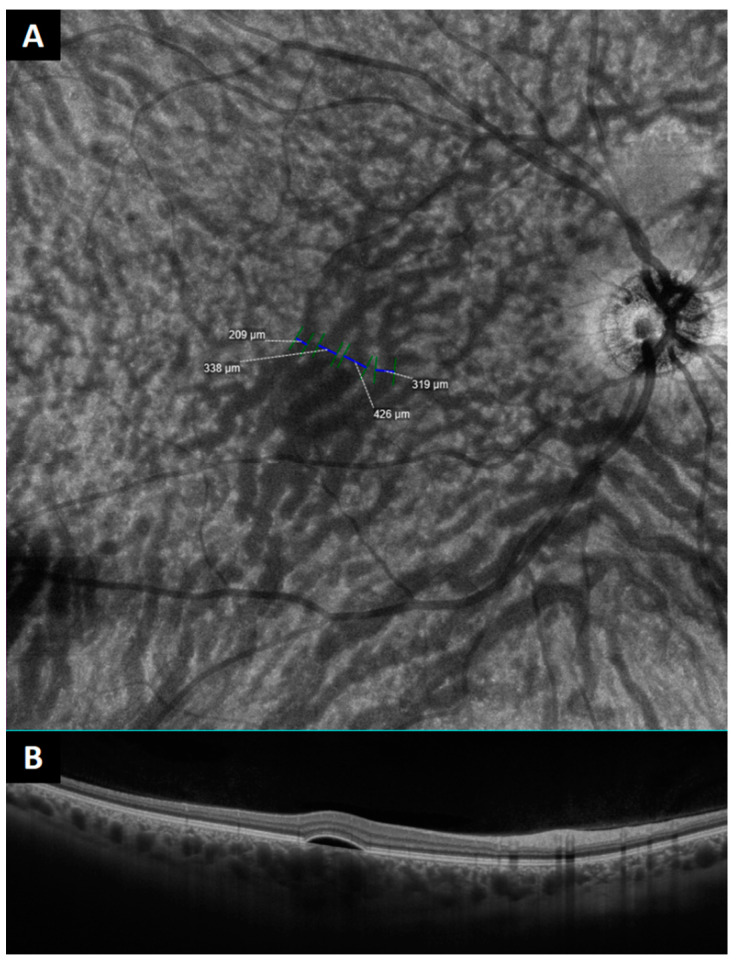
(**A**) En face optical coherence tomography of the macular region showing large anastomoses in a patient affected by central serous chorioretinopathy (CSCR). (**B**) B-scan showing the pachyvessels on the choroid in the central area.

The main variable was the presence of prominent anastomoses in the central macula, which was defined as a connection between the supero-temporal and infero-temporal vortex vein systems, with a diameter ≥150 μm, which crosses the temporal raphe (Figure 1 and Figure 2). As previously defined by Spaide et al., anastomoses was considered to be present if there were two or more anastomotic vessels connecting the adjacent quadrants of vortex veins [20].

**Figure 3:**
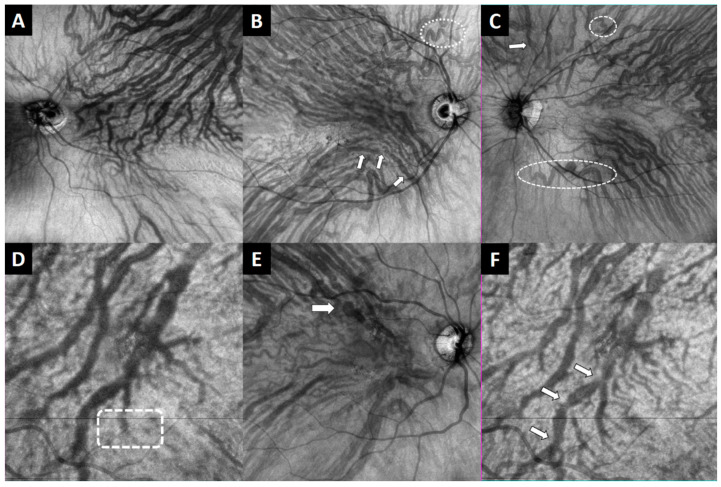
Examples of en face optical coherence tomography images of the choroidal vessel characteristics assessed. (**A**) Asymmetry with predominance of the superotemporal vessels. (**B**) Sausaging of the choroidal vessels (arrow) and corkscrew vessel (dotted circle). (**C**) Corkscrew vessels (dotted circles) and bulbosity (arrow). (**D**) Abrupt termination of a choroidal vessel. (**E**) Bulbosity of a choroidal vessel (arrow). (**F**) Sausaging of a choroidal vessel (arrows).

In addition, the presence of asymmetries in the running pattern of the choroidal vessels relative to a horizontal line across the fovea was assessed. An asymmetry was defined as a predominance in the number or caliber of the supero-temporal or infero-temporal choroidal vessels that cross more than 1000 µm the medial raphe of the macula [15]. The presence of abrupt terminations in the pachyvessels on the EF-OCT images was also assessed (Figure 3) [14].

Lastly, the presence of sausaging, defined by Spaide as three or more contiguous fusiform dilations that vary by at least 50% from the narrowest to largest diameters, and bulbosities, defined as a focal 2× dilation of a blood vessel as compared with the diameter of the surrounding host vessel, were registered [16]. The presence of vessels twisted into a spiral shape, known as corkscrew vessels, was also noted (Figure 3) [21].

EF-OCT images were reviewed in a masked fashion by two independent and experienced readers (LLG and DRL), and the agreement between them was analyzed in the first 77 consecutive CSCR and control eyes. Two more readers were advised in case of disagreement (JIFV and FJMM).

### 2.2. Statistical Analysis

The software package SPSS^®^ (Statistical Package for Social Sciences, v25.0; SPSS Inc., Chicago, IL, USA) was used to perform all statistical tests. Quantitative parameters were provided as the mean and standard deviation. Qualitative values were expressed as their frequency distributions. To assess the inter-rater reliability, the kappa statistic was calculated. Analysis of variance (ANOVA) was applied among the three groups to determine if there were differences. Furthermore, differences between groups were assessed by *t*-test for independent samples and a chi-square test for qualitative variables for paired comparison. The significance level was set at *p* < 0.05.

## 3. Results

The mean age of CSCR patients was 51 ± 10.7 years with 72.2% males, with the mean age of controls being 49.5 ± 14.2 years with 69.5% males (*p* ≥ 0.315).

Among the 135 eyes with unilateral chronic CSCR (with persistent SRF), 79.2% (107 eyes) showed prominent anastomoses in the central macula between the infero-temporal and supero-temporal vortex vein systems, being more frequent than in the unaffected fellow eyes, in which 51.8% of these patients had anastomoses (defined as two or more) (*p* < 0.001). However, no differences were observed between the eyes of CSCR patients and the control group (58.2%; *p* = 0.068) (Table 1).

**Figure 4:**
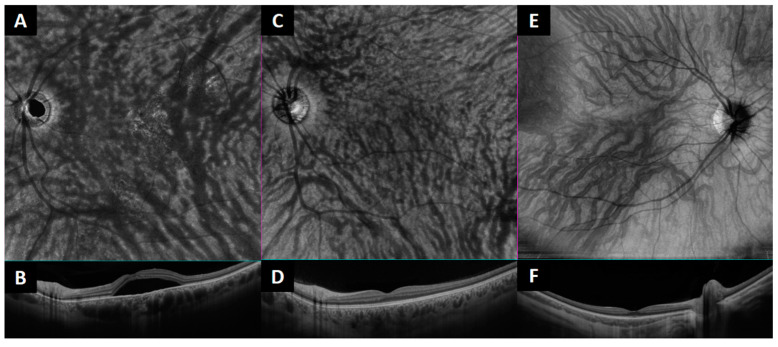
En face optical coherence tomography and the corresponding B scan of the macular region to analyze the presence of intervortex venous anastomosis. (**A**,**B**): example of an eye affected by central serous chorioretinopathy (CSCR). (**C**,**D**): Fellow unaffected CSCR eye. (**E**,**F**): example of a healthy control.

Regarding the number of macular anastomoses, anastomotic connections were more common in the affected CSCR group (2.9 ± 1.8) than in the unaffected fellow eye group (2.1 ± 1.7; *p* < 0.001) and in controls (1.5 ± 1.6 respectively; *p* < 0.001) (Table 1, Figure 4).

Good inter-rater agreement to determine the presence and number of the macular anastomoses was observed, with a kappa index ≥0.84 for CSCR and control eyes (Table 2).

An asymmetry in the distribution of the choroidal vessels was more frequent in 17% of the CSCR eyes than in the unaffected fellow eyes (8.9%) and controls (1.8%) (*p* < 0.001), observing a similar predominance between the supero-temporal and infero-temporal vessels in 11 and 12 CSCR eyes, respectively (Table 3, Figure 3).

An abrupt termination of the pachyvessels on the EF-OCT images was observed in 7.4% of the CSCR eyes, being more frequent than in the unaffected fellow eyes (2.9%) and in healthy subjects (1%) (*p* < 0.001), while observing no differences between fellow eyes and controls (*p* = 0.864) (Figure 3).

Sausaging and bulbosities were present in 23.7% and 22.9% of the CSCR eyes, 7.4% and 14.8% of the unaffected fellow eyes, and 8.1% and 7.3% of the controls, observing no differences among groups (*p* ≥ 0.129). However, corkscrew choroidal vessels were more frequent in CSCR eyes than in the other groups (*p* ≤ 0.033) (Figure 3).

## 4. Discussion

New hypotheses on the physiopathology of the choroidal changes in CSCR patients have been described in recent years, these being the presence of pachyvessels and macular choroidal anastomoses that are remarkably characteristic. In a recent study, Spaide et al. found good agreement between the pachyvessels on B-scan and EF-OCT images, and affirmed that the large anastomosis observed crossing the horizontal raphe in the macula in their study might have been considered as pachyvessels in previous reports [20].

In the present study, intervortex venous anastomosis in the macular area were common in CSCR patients using EF-OCT. This anatomical variation was more frequently observed in the affected eyes of CSCR patients than in the fellow unaffected eyes and healthy controls. Dansingani et al., using EF-OCT, stated that there were pachyvessels traversing the focus of disease in eyes with what they termed pachychoroid spectrum disorders [14]. Nonetheless, they did not specifically mention if this was in the macula or across a watershed zone, and they did not mention anastomoses.

Our findings are also in line with those reported by Spaide et al., who reviewed the ICGA images of 24 eyes with CSCR and found that macular anastomotic connections were more common in these patients compared to healthy controls (*p* < 0.001). The authors also described that, while patients with CSCR showed prominent anastomotic connections primarily centered in the macular region, patients with peripapillary pachychoroid syndrome presented them along the peripapillary region [20]. Interestingly, Ramtohul et al. have observed that the identification of pachyvessels crossing the choroidal watershed zones showed an excellent correlation between en face ultra-widefield (UWF) and OCT UWF ICGA images, showing distinctive features of choroidal venous insufficiency or choroidal congestion with both techniques [22].

Spaide et al. proposed that these anastomoses should be considered as present if there were two or more vessels connecting adjacent quadrants of vortex veins [20]. Our results support this consideration, as 45.4% of healthy controls showed one anastomotic vessel in one of their eyes, or in other words, 28.2% of healthy eyes have at least one anastomosis. Shiihara et al. used EF-OCT images, as in the present study, to quantitatively assess the diameter and running pattern of choroidal vessels in 41 eyes with CSCR, 41 fellow eyes, and 41 healthy controls [15]. In agreement with our results, Shiihara et al. observed the presence of pachyvessels in 31.7% of the normal eyes, as compared to 82.9% in CSCR eyes.

Regarding the definition criteria of anastomoses and pachyvessels, Spaide et al. proposed that these connecting vessels had to have a diameter equal to or greater than that of the retinal arcade vein at the border of the optic disc, which has been described to be around 120 µm [20]. Shiihara et al.,, determined a cut-off mean vessel diameter value between CSCR and normal eyes of 153 µm, having a sensitivity and specificity of 82.9% and 68.3%, respectively. In fact, they observed that eyes with CSCR had larger dilated choroidal vessels as compared with both the fellow eyes of CSCR patients and with healthy controls, the mean vessel diameter being 185 ± 39 µm in CSCR eyes, 171 ± 38 µm in fellow eyes, and 144 ±20 µm in controls, with significant differences among groups (*p* ≤ 0.042). For this reason, we only considered those anastomotic vessels with a diameter greater than 150 µm.

On the other hand, Spaide et al. proposed that there may be a subpopulation of patients with pachychoroid-related abnormalities that do not have venous intervortex anastomoses [20]. The results in the present study are in accordance with this idea, because a fifth of the chronic CSCR patients (20.8%) did not have any macular choroidal anastomosis.

Furthermore, asymmetries in choroidal anastomoses were evaluated. The presence of asymmetrical vessels running in Haller’s layer was more frequently detected in CSCR eyes and fellow eyes than in controls (17% versus 8.9% versus 1.8%), observing no predominance of the supero-temporal or infero-temporal vessels in CSCR eyes. In Shiihara et al.’s study, which had a highly refined automatic method, the symmetry index was 59.4 ± 5.8% in controls, 55.3 ± 7.2% in fellow eyes, and 53.7 ± 6.0% in CSCR eyes. No differences between the CSCR eyes and its fellow eye were noted (*p* = 0.568), although it was significantly smaller in the CSCR and fellow eyes compared to normal eyes (≤0.007). The authors suggested that the existence of a watershed zone in the subfoveal region in CSCR and its fellow eye is less likely than in normal eyes. Differences between Shiihara et al.’s study and the present one includes a smaller number of CSCR patients (41 vs. 135), and all included subjects were Japanese, which is in contrast with ours, in which all subjects were Caucasian. Furthermore, they used a 7 × 7 mm exploration field instead of a 12 × 12 mm one, and the scanning speed was 100,000, while it was 200,000 scans/sec in the present one. Recently, Terao et al. described, using also en face OCT, that the asymmetric dilated vortex vein is a common finding in patients with CSC, being associated with certain biometric factors such as short axial length [23].

Abrupted terminations were also investigated, as Dansingani et al. stated that unlike physiologic vessels, pachyvessels do not narrow at their distal extent, but appear to terminate abruptly [14]. We observed that abrupted termination of the pachyvessels on the EF-OCT images was more frequently observed in the CSCR eyes (7.4%), than in the unaffected fellow eyes (2.9%) and in healthy subjects 1%. On the contrary, Spaide et al. did not observe an abrupt termination of any large vein in any eye using ICGA (20). In our study, when we analyzed the scan cube of the choroidal vessels with an abrupt termination and followed its direction and course, we could observe that these vessels at some point changed to a vertical trajectory. We hypothesize that this could be the actual reason for the apparent abrupt termination, this probably being an artefact, since any vessel in the body becomes thinner at its terminal end unless there is an obstruction.

Recently, variations in the venous caliber, such as sausaging and bulbosities, have been described in eyes with CSCR using widefield ICGA, and could be associated with pathophysiologic alterations related to increased pressure within and remodeling of the larger choroidal veins [16]. In a study including 73 eyes of 41 patients, the sausaging of vessels was seen in three quadrants per eye, the use of corticosteroids being the only significant risk factor. They also described a total of 39 bulbosities in 26 eyes (35.6%), preferentially involving intervortex venous anastomoses. In the present study, sausaging and bulbosities were frequent in CSCR eyes (23.7 and 22.9%), but this vessel appearance was also observed in the fellow and control eyes, with no differences between groups (*p* ≥ 0.129), but with a greater tendency to the existence of the latter in CSC eyes than in fellow eyes and controls. However, one explanation for the lower number of bulbosities in our study could be the limited macular area scanned in contrast with the evaluation of the four quadrants by ICGA done by Spaide et al. Moreover, it should be highlighted that the presence of corkscrew vessels was significantly higher in CSCR eyes than in fellow eyes and controls, this possibly being related to an impaired or altered vascular flow, which subsequently resulted in the appearance of this abnormality.

This is the first study to employ EF-OCT in a large population of CSCR patients to study choroidal macular anastomoses, as well as other choroidal vessel characteristics such as asymmetries, abrupt terminations, vascular caliber variations like sausaging and bulbosities, or a corkscrew appearance. These findings could have important implications in the understanding of pachychoroid disease. Nevertheless, future studies are warranted to analyze whether changes in these anastomotic vessels occur over time, trying to detect thickening and evolution along with the chronicity of the disease, and to evaluate if these anastomoses are formed in the conversion from acute to chronic forms or are already present and to what degree. In this regard, Shiihara et al. have described that it was difficult to determine whether the choroidal vessels were enlarged or congenitally large in CSCR eyes. Hosoda et al. have previously described that genetic factors, specifically CFH and VIPR2 genes, could be associated with the larger vessel choroidal diameter Haller’s layer since birth and after the presence of CSCR [24]. Matsumoto et al. performed a very interesting study creating a monkey model of vortex vein congestion by ligating two vortex veins, and they could observe pachychoroid-related findings, indicating that vortex vein congestion is involved in the pathogenesis of the pachychoroid. Nevertheless, the authors noted that the remodeling of the choroidal drainage route via intervortex venous anastomosis appeared to compensate for the vortex vein congestion created in this model [25]. In the future, it would also be interesting to observe if the cut-off value of 153 µm proposed by Shiihara et al. can predict the likelihood of developing CSC. Finally, as Spaide et al. posited, the detection of anastomosis may offer a method to classify subtypes of pachyvessels and their associated diseases [20].

The present study has several limitations. First, the initial approach to these images is a manual quantification of the number of anastomoses or the presence of vessel abnormalities. However, good inter-rater agreement was observed (kappa index ≥ 0.84). In addition, it is sometimes difficult to achieve a correct segmentation of Haller’s layer and that the segmentation often needs to be manually adjusted. Therefore, algorithms to perform an automated segmentation of en face scans such as the one described by Shiihara et al. are needed to assess the large choroidal vessels [26]. However, algorithms should be improved to reliably analyze the high variability appearance of pathological choroids, as well as to analyze and quantify new features, including variations in the venous caliber, such as sausaging and bulbosities, or the presence of corkscrew vessels [16]. In addition, it should be noted that the entire vortex vein drainage area as well as the number or asymmetries in the latter were not assessed in the present study due to the limited scan size of the OCT device. Lastly, ICGA images of sausaging and bulbosities were not available for all the patients to compare the findings between this technique and en face OCT.

## 5. Conclusions

Choroidal anastomoses between the vortex veins in the macular area were common in CSCR. This anatomical variation was observed more frequently in the affected eyes of patients with CSCR than in the unaffected fellow eyes and in healthy controls. This finding could have important implications regarding the etiopathogenesis and classification or severity of the disease, and could even lead to new therapeutic proposals to reduce choroidal stasis.

## Figures and Tables

**Table 1:** Results of the presence and number of choroidal anastomosis assessed by En Face optical coherence tomography in the three groups studied.

Group	% Patients with Anastomosis	Nº Macular Anastomosis
1. Chronic unilateral CSCR (persistent SRF) (N = 135)	79.2% (107/135)	2.9 ± 1.8 (0 to 8)
2. Fellow unaffected eye (N = 135)	51.8% (70/135)	2.1 ± 1.7 (0 to 8)
3. Controls (N = 110 eyes, 57 patients)	58.2% (64/110)	1.5 ± 1.6 (0 to 6)
*p*-value ANOVA	<0.01 *	<0.01 *
*p*-value (paired comparison)	1 vs. 2: <0.01 *	1 vs. 2: <0.01 *
	1 vs. 3: <0.01 *	1 vs. 3: <0.01 *
	2 vs. 3: 0.068	2 vs. 3: <0.01 *

*: statistically significant.

**Table 2:** Results of the kappa value for the inter-rater macular anastomoses assessment.

Parameter	Group Studied (n = 77 Eyes)	Kappa Value
Qualitative assessment: presence or absence of anastomoses	CSCR eyes	0.847
	Control eyes	0.860
Quantitative assessment: number of anastomoses	CSCR eyes	0.922
	Control eyes	0.848

**Table 3:** Summary of the main characteristics of the choroidal vessels assessed by En Face optical coherence tomography.

Group	Asymmetries (%)	Abrupt Choroidal Vessels (%)	Sausaging (%)	BULBOSITIES (%)	Corkscrew Vessels (%)
1. Chronic unilateral CSCR (persistent SRF) (N = 135)	17% (23/135) 11 superior and 12 inferior predominance	7.4% (10/135)	23.7% (32/135)	22.9% (31/135)	15.5% (21/135)
2. Fellow unaffected eye (N = 135)	8.9% (12/135) 9 superior and 3 inferior predominance	2.9% (4/135)	7.4% (10/135)	14.8% (20/135)	6.6% (9/135)
3. Controls (N = 110 eyes, 57 patients)	1.8% (2/110) 1 superior and 1 inferior predominance	1% (1/110)	8.1% (9/110)	7.3% (8/110)	4.5% (5/110)
*p*-value ANOVA	<0.001 *	0.028	0.073	0.105	0.03
*p*-value (paired comparison)	1 vs 2: <0.001 *	1 vs 2: <0.001 *	1 vs 2: 0.206	1 vs 2: 0.195	1 vs 2: 0.015 *
	1 vs 3: <0.001 *	1 vs 3: <0.001 *	1 vs 3: 0.311	1 vs 3: 0.129	1 vs 3: 0.293
	2 vs 3: <0.001 *	2 vs 3: 0.864	2 vs 3: 0.434	2 vs 3: 0.591	2 vs 3: 0.547

*: statistically significant.

## Data Availability

Data used to support the findings presented in this study are available on request from the corresponding author.
